# A Surgical Strategy for Three-Layer Structure Reconstruction in Total Nasal Defect

**DOI:** 10.3390/jcm15145459

**Published:** 2026-07-13

**Authors:** Bao-Fu Yu, Jiao Wei, Chuan-Chang Dai

**Affiliations:** Department of Plastic and Reconstructive Surgery, Shanghai Ninth People’s Hospital, Shanghai Jiao Tong University School of Medicine, Shanghai 200025, China

**Keywords:** total nasal defect, rhinoplasty, reconstruction, three-layer structure

## Abstract

**Background/Objectives**: Total nasal reconstruction has long represented a formidable surgical challenge. To date, no universally accepted, evidence-based protocol for nasal reconstruction exists to guide clinical practice. This study introduces a novel technique for comprehensive, three-layer nasal reconstruction. Specifically, the approach entails (1) reconstruction of the nasal mucosal lining using a free radial forearm flap; (2) provision of robust structural support via an exogenous extended framework; and (3) restoration of the external nasal skin using an expanded forehead flap. **Methods**: Ten patients underwent reconstruction for full-thickness nasal defects, all achieving successful structural and functional restoration. All surgical procedures were completed successfully, with operative durations ranging from 6.5 to 10.5 h. One patient developed an infection involving the rib cartilage graft. Following thorough debridement, the radial forearm free flap healed uneventfully. A second patient experienced postoperative vascular compromise of the flap. Intraoperative exploration revealed inadequate perfusion; immediate microsurgical revision—including adjustment of recipient vessels and/or re-anastomosis—successfully restored flap viability. **Results**: Primary wound healing was achieved in all patients within 10–22 days. All patients completed a follow-up of 12–36 months (mean: 21.5 months). Both patients and the surgical team rated postoperative nasal aesthetics as satisfactory. Objective functional assessments—including anterior rhinomanometry and peak nasal inspiratory flow—demonstrated no clinically significant impairment in nasal airflow. **Conclusions**: This surgical strategy for reconstructing the three-layer nasal architecture in patients with total nasal defects represents a rational and clinically viable approach—offering a valuable reference for rhinoplasty surgeons performing such complex reconstructions.

## 1. Introduction

Total nasal defects may result from trauma, post-surgical tumor resection, or other etiologies [1]. Their reconstruction remains a formidable clinical challenge, marked by exceptional technical complexity. Successful reconstruction of a total nasal defect necessitates the restoration of three anatomically and functionally distinct layers: the internal nasal mucosal lining, the cartilaginous skeletal framework, and the external cutaneous coverage [2,3]. Crucially, preservation of unobstructed nasal ventilation must be rigorously prioritized throughout the reconstruction of this tri-layered architecture [4,5]. Collapse or stenosis of the internal lining—whether due to inadequate structural support or poor tissue viability—may impair airflow, thereby significantly compromising respiratory function and the patient’s quality of life [6,7,8]. To date, no universally accepted, evidence-based reconstruction protocol exists to guide clinical practice.

We previously described the application of exogenous extension frameworks in nasal repair and reconstruction [9]. These frameworks operate independently of the native nasal septal support. Building on our accumulated clinical experience, this study introduces a novel technique for comprehensive three-layer nasal reconstruction. Specifically, the approach entails: (1) using a free radial forearm flap to reconstruct the nasal mucosal lining; (2) employing an exogenous extension framework to provide robust structural support; and (3) utilizing an expanded forehead flap to restore the external nasal skin.

## 2. Methods

This study is a retrospective case study and the ethical approval was waived. It was conducted in accordance with the principles of the Declaration of Helsinki. This report presents a surgical technique performed at a single institution. Informed consent was obtained from all participants prior to their inclusion in the study. Preoperative antibiotics were administered as part of the standard protocol.

All 10 patients treated between 2020 and 2025 were consecutive patients diagnosed with full-thickness total nasal defects who met inclusion criteria: complete loss of nasal skin, mucosa and cartilaginous framework; no severe systemic comorbidities contraindicating long-duration general anesthesia and microsurgery. Exclusion criteria: uncontrolled diabetes, peripheral vascular disease, active local facial malignancy or infection, inability to complete regular long-term follow-up. No eligible consecutive patients were excluded from this retrospective series.

**Figure 1:**
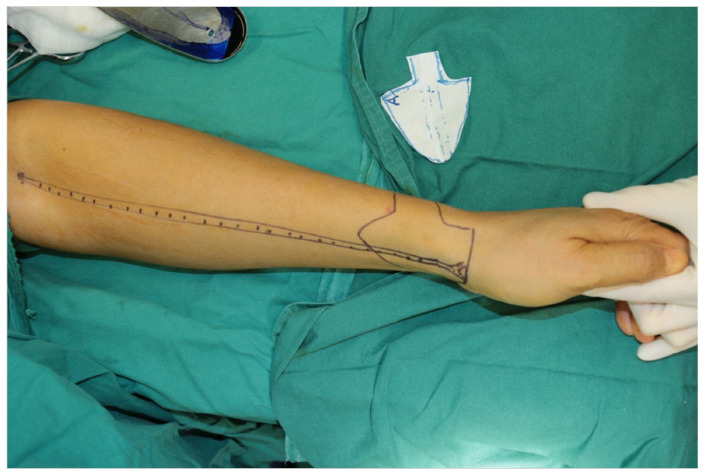
This free flap was harvested with the radial artery and its paired radial venae comitantes serving as its vascular pedicle.

This procedure was performed under general anesthesia. For reconstruction of the nasal lining, a free skin flap was designed on the forearm based on the three-dimensional contour of the nasal defect, following nasal debridement and scar excision to prepare the recipient site. This free flap was harvested with the radial artery and its paired radial venae comitantes serving as its vascular pedicle (Figure 1). After flap elevation, the donor site was managed either by primary closure via direct suturing or by coverage with a split-thickness skin graft, depending on the size and tension of the wound.

**Figure 2:**
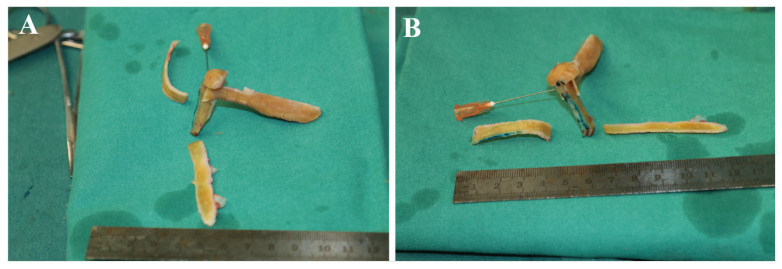
Fabrication of autologous rib cartilage scaffold. (**A**) Mushroom-shaped and willow-leaf-shaped rib cartilage segments assembled via mortise-and-tenon joint for nasal columella and dorsal support; (**B**) Two rectangular rib cartilage strips prepared for bilateral alar cartilage reconstruction.

We harvested the sixth rib cartilage from the patient and sculpted it into a mushroom-shaped segment and a willow-leaf-shaped segment, which were joined using a mortise-and-tenon joint. In addition, two rectangular strips of rib cartilage were prepared for alar cartilage reconstruction (Figure 2).

**Figure 3:**
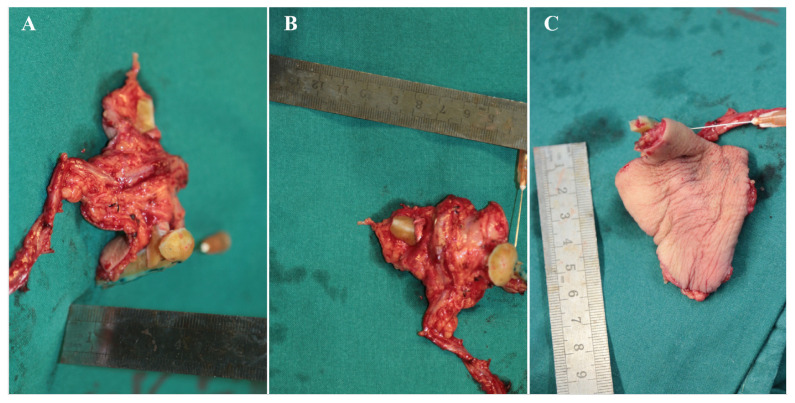
Stepwise assembly of radial forearm free flap and costal cartilage framework. (**A**) Willow-leaf cartilage graft placed subcutaneously under the lining flap for nasal dorsal support; (**B**) Mushroom-shaped cartilage graft fixed centrally for columellar support; (**C**) Two rectangular cartilage strips sutured bilaterally inside the flap to reconstruct alar cartilages.

**Figure 4:**
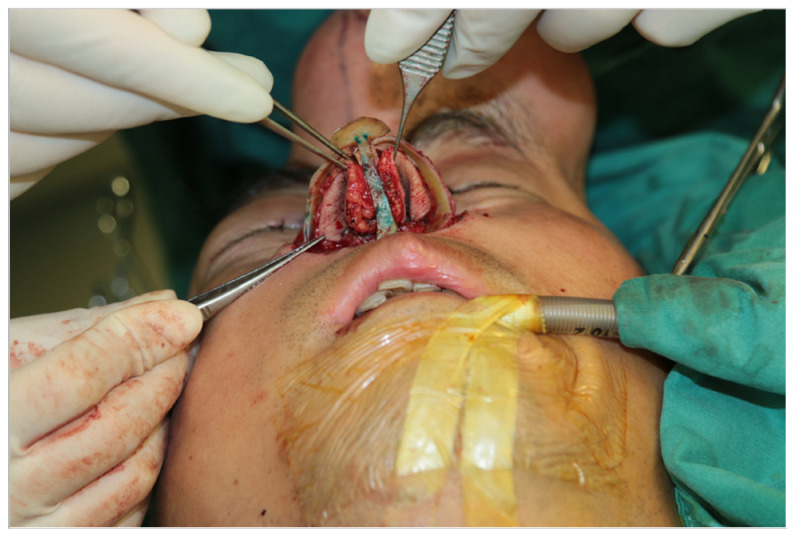
Intraoperative photographs demonstrate the transplantation of both the nasal scaffold and a free skin flap to reconstruct the nasal cavity lining and provide structural support.

The trimmed costal cartilage scaffold and the excised free flap were meticulously assembled. A willow-leaf–shaped costal cartilage segment was placed subcutaneously beneath the free flap, whereas a mushroom-shaped costal cartilage graft was implanted to reconstruct the support of nasal columella. Two rectangular costal cartilage grafts were positioned within the flap to restore the nasal alae (Figure 3). Following complete assembly of the flap and costal cartilage scaffolds, the composite construct was transplanted into the nasal recipient site. An intraoperative photograph is shown in Figure 4.

The facial artery and paired facial venae comitantes were routinely selected as recipient vessels for microvascular anastomosis. After preparation of the nasal recipient bed, the radial artery of the free forearm flap was end-to-end anastomosed to the facial artery under a 10× surgical microscope; radial venae comitantes were anastomosed to facial venae comitantes with 9-0 nylon microsutures. Vascular patency was confirmed by bright capillary refill of the flap margin and unobstructed venous outflow before composite scaffold–flap complex implantation.

**Figure 5:**
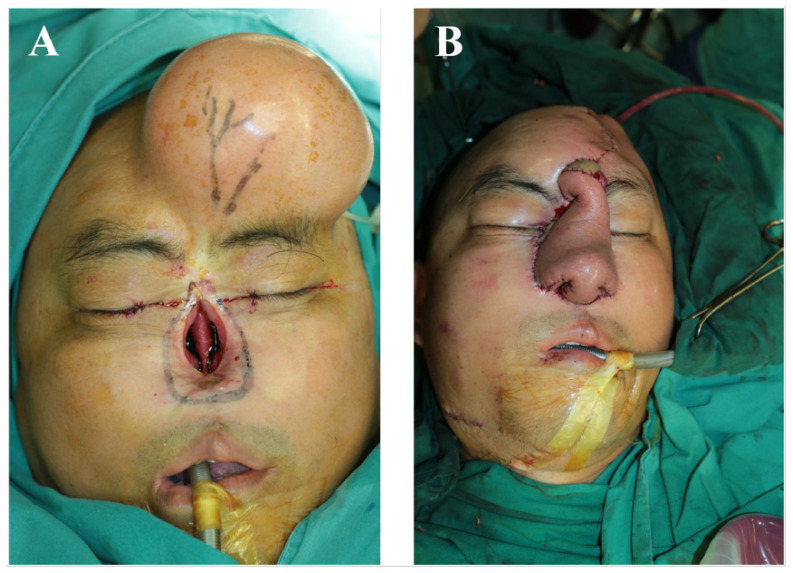
Nasal lateral skin reconstruction was performed using a forehead-based expanded flap for transplantation. (**A**) Surgical design marking diagram; (**B**) After the expander was removed, the forehead flap was transplanted onto the nose to repair the defect.

For reconstruction of the external nasal skin, our approach aligns with previously reported techniques utilizing forehead flap transfer [10,11,12]. Specifically, we performed tissue expansion via forehead implantation of a tissue expander, with the expanded flap vascularized by the supratrochlear artery (a branch of the ophthalmic artery) and vein as its pedicle. Initially, a tissue expander was implanted in the forehead. Subsequently, normal saline was gradually injected to expand the device until the target volume was achieved. Thereafter, the expander was surgically removed, and the expanded forehead skin flap was transposed to reconstruct the external nasal skin defect (Figure 5). The donor site was primarily closed with direct suturing. The forehead-expanded flap was based on the supratrochlear artery and its accompanying veins. At the nasofrontal pivot point, limited subcutaneous debulking was performed on the distal undersurface of the flap to prevent contour irregularities—particularly nasal tip fullness—while preserving the integrity of the subdermal vascular plexus to ensure adequate flap perfusion. The subcutaneous fat at the pivot site was partially excised, but no separate skin graft was used to cover the exposed undersurface. Instead, adjacent forehead soft tissue was carefully undermined and advanced to achieve primary closure of the raw pivot area prior to flap transposition onto the nasal dorsum.

Postoperative Management: External nasal splint fixation was maintained for 2 weeks to preserve nasal contour. Intranasal silicone stents were placed for 4 weeks to support the lining and prevent contracture. No nasal prosthesis (e.g., retainer) was used in any patient. Wound care, antibiotic prophylaxis, and anti-swelling treatment were administered routinely.

Outcome Assessment: The Rhinoplasty Outcome Evaluation (ROE) questionnaire is a validated tool mainly designed for standard rhinoplasty patients with partial nasal deformity, not patients with complete full-thickness total nasal loss. Most ROE items focus on minor nasal tip/dorsal deformities, which cannot fully reflect the unique aesthetic and functional concerns of patients with total nasal absence. Therefore, we adopted a unified 5-point global satisfaction scale for both patients and independent surgeons to comprehensively evaluate overall nasal morphology, symmetry, contour and matching with facial subunits. (1) Aesthetic Evaluation: Patient satisfaction: Anonymous in-person survey using a 5-point scale (1 = very dissatisfied, 5 = very satisfied) at follow-up visits. Surgeon evaluation: Three independent senior plastic surgeons (not involved in surgery) rated aesthetic outcomes using the same 5-point scale. (2) Functional Evaluation: Anterior rhinomanometry and peak nasal inspiratory flow (PNIF, a quantitative parameter reflecting maximum nasal inspiratory airflow during voluntary deep inhalation) were measured by two independent technicians (blinded to study design) preoperatively and at final follow-up. Mean values and 95% confidence intervals were calculated. Because the patient presented with a complete nasal defect, rhinomanometry and PNIF testing were not performed due to practical constraints.

## 3. Results

From 2020 to 2025, a total of 10 patients underwent this reconstructive procedure and achieved successful restoration of full-thickness nasal defects using this integrated repair strategy. The cohort comprised six male and four female patients, with a mean age of 48 years (range: 27–68 years). Baseline and clinical data of 10 patients are shown in Table 1. All surgeries were completed successfully, with operative durations ranging from 6.5 to 10.5 h. One patient developed a postoperative infection involving the rib cartilage graft. In this case, the graft was surgically excised and temporarily buried beneath the scalp for biological stabilization. Following thorough surgical debridement, the radial forearm free flap healed without complication. Three months later, the rib cartilage scaffold was retrieved and re-implanted into the nasal defect to complete definitive reconstruction. A second patient experienced postoperative vascular compromise in the flap. Intraoperative exploration confirmed compromised perfusion, and immediate microsurgical intervention—comprising adjustment of recipient vessels and re-anastomosis—successfully restored flap viability. Primary wound healing was achieved in all patients within 10–22 days postoperatively.

All patients completed a follow-up period of 12–36 months (mean: 21.5 months). Aesthetic satisfaction: The mean patient-reported score was 4.6 ± 0.3; the mean surgeon-assessed score was 4.5 ± 0.4. Nasal ventilation: Postoperative rhinomanometry and PNIF values fell within established normal reference ranges. Mean postoperative nasal airway resistance at final follow-up was 48.2 ± 10.5 Pa·s/mL (normal reference: <50 Pa·s/mL). Mean postoperative PNIF was 142.6 ± 20.4 L/min (normal reference: >120 L/min). All postoperative functional measurements remained within published normative ranges for healthy adults, confirming absence of clinically relevant airflow obstruction.

**Figure 6:**
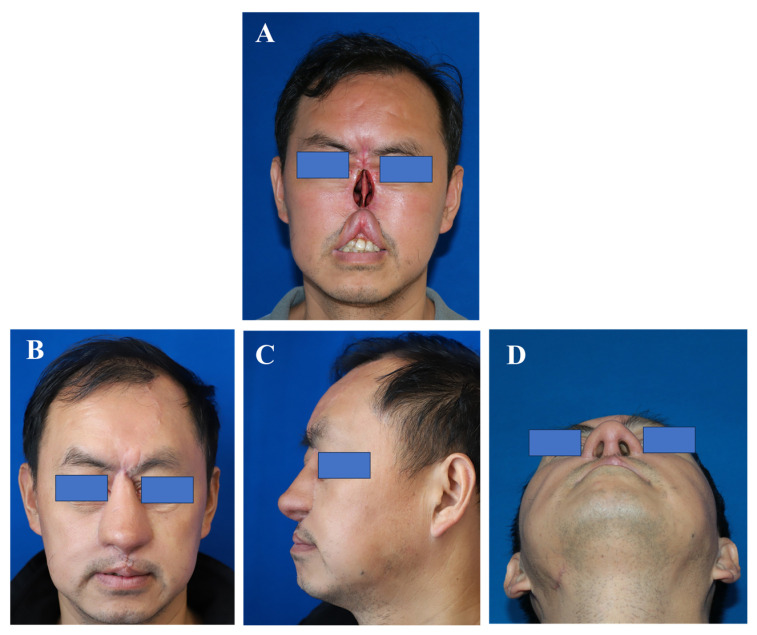
It presents a representative case managed using this technique, demonstrating favorable long-term aesthetic and functional outcomes.(**A**) A frontal photo of the patient’s nose before the surgery; (**B**) Post-operative patient’s frontal view of the nose photo; (**C**) Post-operative photos of the patient’s side of the nose; (**D**) Post-operative photos of the nasal base position of the patient.

Figure 6 illustrates a representative case treated with this technique, demonstrating favorable long-term aesthetic and functional outcomes.

## 4. Discussion

Total nasal reconstruction has long posed a formidable surgical challenge [4,5]. The precise design and elevation of soft-tissue flaps for nasal resurfacing—coupled with the concurrent construction of a robust, load-bearing structural framework—are both essential and technically demanding [13,14,15]. The structural framework fulfills a pivotal biomechanical function: it counteracts flap contracture and supports accurate three-dimensional reconstruction of the nasal subunits. Furthermore, nasal lining flaps—particularly those lacking intrinsic rigidity—are susceptible to inward collapse into the nasal cavity, thereby compromising airway patency and ventilatory efficiency [11,16,17]. Critically, the absence of an intact nasal septum severely limits the surgeon’s ability to establish a stable, biomechanically competent framework that concurrently meets aesthetic and functional requirements [18,19,20]. Given the paucity of comprehensive, technique-oriented reports in the contemporary literature, there remains an urgent need for innovative reconstructive strategies and rigorous clinical validation.

In previous studies, Gary described a series of 10 consecutive patients who required reconstruction of the nasal vestibular and columellar lining [4]. In most cases, reconstruction also extended to the nasal floor—the anatomical platform supporting the alar bases and columella—as well as to defects involving adjacent facial aesthetic subunits. Aesthetic nasal reconstruction was performed using two distinct skin paddles to restore the nasal vestibular and columellar lining, an artistically sculpted cartilaginous framework, a paramedian forehead flap for external coverage, and supplementary local flaps or grafts to address concomitant defects in neighboring facial units. We acknowledge that this study has yielded promising therapeutic outcomes. In the previously reported treatment protocol, surgical reconstruction was performed in two staged procedures: the first involved transplantation of a free forearm flap with concomitant full-thickness skin grafting; the second comprised implantation of a rib cartilage scaffold and transfer of an expanded forehead flap. In contrast, our refined reconstructive strategy integrates all essential components—including free flap transplantation, rib cartilage scaffold implantation, and forehead flap transfer—into a single-stage operation, thereby obviating the need for full-thickness skin grafting. Moreover, the simultaneous use of a free flap and a structural rib cartilage scaffold affords robust mechanical support, effectively minimizing postoperative flap retraction—a complication frequently observed in Asian patients owing to inherent anatomical and tissue-specific characteristics. Cheng-I et al. reported the use of various free flaps—including medial sural artery perforator (MSAP) flaps, anterolateral thigh (ALT) flaps, and a radial forearm flap—in conjunction with forehead expansion flap surgery for simultaneous nasal reconstruction, with favorable clinical outcomes documented [5]. However, based on our accumulated clinical experience, ALT flaps—owing to their relatively greater subcutaneous thickness—are associated with a higher incidence of postoperative ventilatory compromise [21,22]. Consequently, we do not recommend their routine use in nasal reconstruction. Furthermore, this study did not incorporate concurrent rib cartilage grafting as structural support. Given the absence of such scaffolding, the procedure may offer limited or no prophylactic benefit against postoperative flap retraction. In contrast, our clinical data demonstrate that simultaneous implantation of autologous rib cartilage scaffolds significantly mitigates flap retraction, particularly in Asian patients. Thus, we believe that our proposed repair strategy has a very good complementary value to the aforementioned repair strategies.

We have developed an exogenous extension framework composed of expanded polytetrafluoroethylene integrated with autologous cartilage for rhinoplasty, yielding satisfactory clinical outcomes. Building upon these results, we adapted the framework for total nasal reconstruction, hypothesizing that it would effectively overcome the challenges inherent in complete nasal septal defects [9,10]. Because this framework provides structural support independently of the native nasal septum, it is particularly well suited for patients with total nasal defects. Precise assembly of the framework with the nasal lining flap is critical: inadequate support predisposes the lining flap to collapse into the nasal cavity. To prevent such collapse—and thereby preserve optimal nasal ventilation—the framework is inserted into the superficial dermal layer. Prior clinical studies have predominantly focused on external nasal morphology; however, late intranasal structural changes following reconstruction remain poorly characterized. To address this knowledge gap, we specifically evaluated intranasal structural stability and systematically optimized the stent–flap assembly configuration. Consequently, we developed an innovative exogenous extension framework and established a rational, reproducible protocol for its integration with the lining flap. For reconstruction of the external nasal skin, we employed the forehead flap transfer technique—a well-established approach consistently reported in the literature for nasal reconstruction.

Building upon our prior clinical experience, we herein introduce a three-layer reconstruction technique. This approach comprises: (i) reconstruction of the nasal lining using a free radial forearm flap; (ii) provision of robust structural support via an exogenous extension framework; and (iii) coverage of the external nasal surface with an expanded forehead flap. In clinical application, this reconstructive strategy has consistently restored both nasal structure and function in patients with total nasal defects. Long-term follow-up data demonstrate favorable aesthetic and functional outcomes, along with an acceptable safety profile. Unlike traditional alar spreader grafts and dorsal onlay grafts that rely on residual nasal septum for anchoring, our exogenous extended framework forms an integrated self-supporting three-dimensional complex independent of native septal cartilage. Combined simultaneous implantation with RFFF lining in one-stage surgery effectively prevents lining flap collapse and long-term tissue retraction, which is a key technical improvement compared with previous staged cartilage graft protocols.

In this study, we did not reconstruct the superior lateral cartilage to provide structural support to the nasal lateral wall. The primary rationale is that our implanted scaffold successfully reconstructed three key anatomical components: (1) the lower lateral nasal cartilage (i.e., the alar cartilage), (2) the columellar support, and (3) the dorsal nasal support. Notably, no reconstruction of the nasal septum was performed within the nasal cavity. Consequently, upon entry of the external nasal valve into the nasal cavity, the normally separate bilateral nasal passages merged into a single common airway. As a result, nasal ventilation dynamics differed from those observed preoperatively in the two independent nasal cavities. Clinical follow-up demonstrated satisfactory outcomes with respect to both nasal morphology and ventilatory function—representing a novel aspect of our approach. Nevertheless, compared with conventional techniques involving concurrent reconstruction of lateral nasal wall support, there remains a paucity of evidence regarding the functional and anatomical effects of our strategy. This study constitutes a retrospective case series. Moving forward, we aim to validate these findings through a prospective, controlled trial.

There were several rationale and technical considerations. Temporary subcutaneous banking in the scalp was used to preserve autologous cartilage, maintain viability, and avoid donor-site morbidity from re-harvesting. The infected costal cartilage was immersed in povidone-iodine solution and subsequently buried beneath the scalp for temporary preservation. To our knowledge, this approach has not been previously documented in the literature. Based on our clinical experience, the scalp’s rich vascular supply is the primary rationale for selecting this site. Prior to implantation, the costal cartilage undergoes thorough irrigation and antiseptic treatment with povidone-iodine. This strategy aims to preserve autologous graft material and potentially obviate the need for secondary harvest during subsequent reconstructive procedures. However, this technique remains investigational; current evidence does not support its classification as definitively safe. Notably, we explicitly contraindicate subgaleal burial of infected costal cartilage in cases of Pseudomonas aeruginosa infection, given the organism’s high virulence and propensity for persistent biofilm formation; in such instances, complete discard is strongly recommended. The exogenous extended framework provides sufficient vertical and lateral support; side wall grafts were unnecessary and avoided over-correction.

This study has several limitations that should be acknowledged. First, it was designed as a single-center, retrospective investigation with a relatively small sample size of only 10 patients, which may limit the generalizability of the findings and the statistical power of the analyses. Second, the follow-up period was restricted to within 36 months, with no long-term data available beyond this time point, thereby precluding assessment of very late outcomes or delayed complications. Third, the absence of a control group prevented direct comparative analysis between the proposed technique and conventional multi-stage surgical approaches, limiting the ability to draw definitive conclusions regarding superiority or equivalence. Finally, although aesthetic outcomes were evaluated by independent assessors, subjective judgment remained inherent in such assessments, which may introduce potential variability and bias in the interpretation of cosmetic results. The aesthetic evaluation relied on an unvalidated 5-point global visual analogue scale rather than a standardized ROE questionnaire, which may introduce inter-rater subjective bias. Future prospective studies will combine both global satisfaction scoring and modified total nasal defect-specific ROE items to improve assessment objectivity.

## 5. Conclusions

In conclusion, this surgical strategy for reconstructing the three-layer nasal architecture in cases of total nasal defect represents a rational and clinically feasible approach, providing a valuable reference for rhinoplasty surgeons undertaking such complex reconstructions.

## Figures and Tables

**Table 1:** Baseline and clinical data of 10 patients.

No.	Age	Sex	Etiology	Defect Duration	Defect Range	Follow-Up (mo)	Complications	Revision	Stent Use
1	54	M	Tumor resection	6 mo	Total nose	36	None	No	Yes
2	38	F	Trauma	12 mo	Total nose	28	Flap vascular crisis	Yes	Yes
3	62	M	Tumor resection	3 mo	Total nose	24	None	No	Yes
4	45	M	Trauma	18 mo	Total nose	22	None	No	Yes
5	27	F	Tumor resection	4 mo	Total nose	18	None	No	Yes
6	68	M	Tumor resection	2 mo	Total nose	16	Cartilage infection	Yes	Yes
7	41	F	Trauma	24 mo	Total nose	14	None	No	Yes
8	50	M	Tumor resection	8 mo	Total nose	13	None	No	Yes
9	36	F	Trauma	10 mo	Total nose	12	None	No	Yes
10	59	M	Tumor resection	5 mo	Total nose	12	None	No	Yes

## Data Availability

No new data were created or analyzed in this study.
